# X4 viruses are frequently archived in patients with long-term HIV infection but do not seem to influence the “inflamm-aging” process

**DOI:** 10.1186/1471-2334-13-220

**Published:** 2013-05-16

**Authors:** Annalisa Saracino, Laura Monno, Luigia Scudeller, Giuseppe Bruno, Nicoletta Ladisa, Grazia Punzi, Anna Volpe, Antonella Lagioia, Gioacchino Angarano

**Affiliations:** 1 Clinic of Infectious Diseases, University of Bari, Bari, Italy; 2 Clinic of Infectious Diseases, University of Foggia, v.le L. Pinto 1, Foggia 71100, Italy; 3 IRCCS San Matteo, Pavia, Italy

**Keywords:** HIV proviral DNA, Co-receptor tropism, CXCR4, CCR5, Geno2pheno, Aging, Inflammation markers

## Abstract

**Background:**

Co-receptor tropism (CRT) in patients with a long history of HIV-1 infection and antiretroviral treatment has been rarely investigated to date. The aim of this study was to determine the prevalence of X4 and R5 strains in patients with a >15-year follow-up and to investigate the demographical, viral, immunological, clinical and therapeutic determinants of CRT in this population. The possible influence of CRT on the inflammation state related to chronic HIV infection was also examined.

**Methods:**

A total of 118 HIV-1 infected patients with an initial HIV-1-positive test before 1997, and still on follow-up, were enrolled and consecutively submitted to blood sampling. Of these, 111 were on antiretroviral therapy and 89/111 (80.2%) had a plasma viral load (pVL) <25 copies/ml at testing. HIV-1 DNA was extracted and amplified from PBMCs for *env* gp120 sequencing. CRT was assigned by using geno2pheno and isolates were classified as X4 (FPR ≤20%) or R5 (FPR >20%). Level of serological inflammation biomarkers including IL-6, hsPCR, and D-dimers were measured.

**Results:**

An X4 virus was evidenced in HIV-1 proviral DNA of 50 patients (42%) while the remaining 68 patients were classified as R5. The median follow-up was 19 years (range 15–25). No association was observed between CRT and sex, age, nationality, subtype, HIV risk factor, HBV/HCV co-infection, baseline CD4+ cell count and pVL, overall duration of antiretroviral therapy, past exposure to mono-or dual therapies, and duration of NNRTI or PI-based therapy. The presence of an X4 strain was associated with CD4 nadir (p = 0.005), CD4 absolute count over time (p < 0.001), and cumulative positive (copy/years) viremia (p <0.001) during the whole patient history. No differences were found between R5 and X4 patients regarding inflammation marker levels including Il-6, hsPCR and D-dimers.

**Conclusions:**

An archived X4 virus was demonstrated in 42% of patients with a >15-year-history of HIV infection. This presence was clearly associated with a greater exposure to positive viremia and a poorer CD4 trend over time compared to R5, independent of type and duration of antiretroviral treatment. CRT does not seem to influence the inflammation rate of patients aging with HIV.

## Background

The third variable (V3) region of gp120 is responsible for the differential CXCR4 or CCR5 co-receptor binding to HIV-1, and genotypic methods and bioinformatic tools based on V3 sequencing have been developed to facilitate the prediction of co-receptor tropism (CRT) [1]. The selective pressure leading to emergence of either CXCR4-using (X4) or CCR5-using (R5) variants is complex but its relevance in the HIV pathogenesis and natural history remains only partially understood [2,3]. R5 variants predominate during the acute phase [4], and also during the asymptomatic phase of HIV-1 infection. Whether this R5 predominance reflects a transmission bias [5] or a post-transmission amplification bias (due to a higher proportion of CCR5 + CD4+ T cells recruited during acute HIV-1 infection) [6], rather than a superior in vivo fitness of R5 strains, remains uncertain. During the course of infection, depending on the HIV-1 subtype and usually in concomitance with the earliest signs of disease progression, a proportion of patients experience the emergence of X4 variants [7]. However, this shift is unnecessary for AIDS progression; in fact, it is observed in only about 50% of subtype-B infected patients [8]. Moreover, also the time span for seroconversion to AIDS does not differ significantly between individuals with pure R5 variants and those with non-R5 (pure X4 and dual-mixed, DM) variants [8]. Hence, this shift might be the consequence, rather than the cause, of immunological deterioration which weakens the selective pressure, thus promoting non-R5 variants in vivo. Moreover, it is unknown if X4 variants emerge from latency many years after an initial independent transmission or, more likely, if they evolve from preexisting R5 variants through a progressive acquisition of a broader receptor affinity [9]. Most information is derived from studies of patients evolving to AIDS in the pre-HAART era. If this viral evolution can be modified by antiretroviral therapy and by the consequent immune-reconstitution, is still a point of dispute. When patients initiate antiretroviral therapy (ART), both increased and decreased frequencies of X4 viruses have been reported [10-14]; in addition, even if the presence of CRT does not seem to predict treatment success [15], recent cross-sectional studies have demonstrated X4 strains in 40%-55% of patients with antiretroviral exposure and therapy failure [16-19].

The prevalence of CRT in a population of patients with a lengthy history of HIV-1 infection and antiretroviral treatment has been rarely investigated. These patients, even when administered highly effective ART regimens, are persistently exposed to antigen-stimulation leading to a peculiar condition of chronic inflammation and hypercoagulability, both of which are involved in determining mechanisms of aging [20,21]. In fact, surrogate biomarkers of chronic inflammation/hypercoagulability, such as IL-6, HsPCR and D-dimer, are altered in HIV-infected subjects compared to matched uninfected controls, and correlate with the frequency of both AIDS [22] and non-AIDS events [23-25] and also with overall mortality. Moreover, these biomarker levels are higher in HIV-infected individuals with detectable HIV-1 RNA [23,26]. It is still unknown if X4 and R5 strains can differently affect the process of HIV-related inflammation and aging.

The primary objective of the present study was to determine the prevalence of X4 (non-R5) and R5 strains in patients with HIV infection for more than 15 years and to investigate the possible association of CRT with the demographical, viral, immunological, clinical and therapeutic data. The possible influence of CRT on the inflammation state related to the long-lasting HIV infection was also evaluated by measurement of several plasma surrogate markers.

## Methods

### Patients

All HIV-1 infected patients testing HIV-1 positive before 1997 and still being followed at the Clinic of Infectious Diseases, University of Bari, Italy were eligible for the study. Clinical data were extracted from the database of the institute and included: date of first HIV-positive test, demographic data, information regarding risk factors for HIV acquisition, onset of opportunistic infections (AIDS events), CDC staging (revised 1993), non-AIDS events, blood biochemical parameters, CD4+ cell count, plasma HIV-1 viral load, clinical and therapeutic history and reported side effects. The following diseases were considered as serious non-AIDS events: acute myocardial infarction, congestive heart failure, coronary artery disease requiring drug treatment, coronary revascularization, decompensated liver disease, deep vein thrombosis, diabetes mellitus, end-stage renal disease, non-AIDS cancer, peripheral arterial disease, pulmonary embolism, and stroke [27]. A total of 118 patients were enrolled and, after informed consent, consecutively subjected to blood sampling for genotypic CRT testing. As most patients were expected to have low or undetectable plasma viral load, CRT was determined for all subjects on proviral DNA which furnishes highly comparable results with those from viral RNA [28]. Concurrently, patients were also submitted to routine blood exams for the assessment of immune-virological parameters (CD4+ cell count and HIV viral load) and surrogate markers of inflammation and aging (IL-6, hsPCR, D-dimers).

### Gp120 sequencing and CRT assignment

HIV-DNA was extracted and amplified from PBMCs and gp120 sequencing on proviral DNA was performed as previously described [29]. Only one PCR product per sample was subjected to standard population sequencing. Sequences were analyzed with Seqscape software v2.5 (Applied Biosystems, Foster City, CA). Nucleotide mixtures were considered if the second highest peak in the electropherogram was >25%. CRT was inferred with the geno2pheno[coreceptor] algorithm (http://coreceptor.bioinf.mpi-inf.mpg.de/), setting the false positive rate (FPR) at 20%, and isolates were classified as R5 (FPR >20%) or non-R5 (FPR ≤20%). Only clonal prediction was employed for classifying sequences.

### HIV-1 subtyping

The HIV subtype was assigned by phylogenetic analysis (neighbor-joining method using Kimura two-parameter distances and Simplot analysis) of *pol* (complete protease and reverse transcriptase) (Viroseq HIV Genotyping Kit; Applied Biosystems, Foster City, CA) and *env* sequences.

### Markers of inflammation

Surrogate markers of inflammation and aging in HIV-positive patients, including IL-6, HsPCR, and D-dimers, were quantified on plasma samples coincident with CRT using commercially available assays. As previous reports [30] suggest a role for IL-7 in inducing viral evolution towards X4 viruses in vitro, the level of this interleukin was also measured. Plasma D-dimers were quantified by means of enzyme-linked fluorescent assay (Vidas D-Dimeri Exclusion II, Biomerieux) at the central laboratory of the University-Policlinico Hospital of Bari (normal values: 0–500 ng/ml). HsPCR, IL-6 and IL-7 were measured at the Infectious Diseases Laboratory by commercially available assays (for hs-PCR: Biokit Quantes CRP, normal values: 0–300 ng/ml; for IL-6: Boster Biological Immunoleader, detection limit: 0.3 pg/ml, assay range: 4.69 - 300 pg/ml; for Il-7: IDElisa Human IL-7, Labs Biotechnology, Canada, detection limit: 16 pg/ml, assay range:16–1024 pg/ml, respectively).

### Statistical analysis

Descriptive statistics were produced for all variables. Mean and standard deviation (SD) were calculated for normally distributed variables, and median and interquartile range (IQR) for non-normally distributed variables. The Mann–Whitney test was used to compare X4 and R5 groups in terms of quantitative variables while the Fisher exact test was adopted for categorical variables. Multivariate mixed models were utilized to investigate the relationship between the CD4 slope/VL over time and CRT; models were adjusted for baseline values; interaction between CRT and time was included.

Since inflammation marker measurements are subject to detection limits (particularly lower detection limits), interval regression was used to explore the association of IL-6, IL-7, HsPCR, D-dimers and CRT (and other variables), by means of the intreg command Stata after log transformation (natural logarithm).

Stata computer software version 12.0 (Stata Corporation, 4905 Lakeway Drive, College Station, Texas 77845, USA) was used for statistical analysis.

### Ethics

The research did not require approval from the ethics committee, according to the Italian law, since it was performed as an observational study in the context of normal clinical routines (art.1, leg. decree 211/2003). However, all patients provided informed consent for the use of their data for research purposes. Blood samples were taken as part of standard patient care; DNA samples and data were previously anonymized, according to the requirements set by Italian Data protection Code (leg. Decree 196/2003).

## Results

### Patient characteristics according to CRT

The 118 HIV-1-infected patients were mostly males (74.5%) (88 men, 30 women) with a 19-year (range 15–25) median infection duration (from first HIV-positive testing). One-hundred eleven (94.1%) were on antiretroviral therapy, 89 of whom (80.2%) had plasma viral load (pVL) <25 copies/ml at time of testing.

A total of 50 (42%) patients showed a X4 virus in their HIV-1 proviral DNA while the remaining 68 patients were classified as R5. The patient characteristics according to CRT are summarized in Table 1. No association was observed between CRT and sex, age, nationality, subtype (B versus non-B), risk factor for acquisition of HIV infection, HCV and HBV co-infection. No patient presented an AIDS-related opportunistic infection at the time of the study; the proportion of patients with a previous AIDS diagnosis was similar in the two groups; R5 patients presented a slightly higher frequency of previous or current non-AIDS events but this difference was not statistically significant.

**Table 1:** Patient characteristics according to CRT

	**R5**	**X4**	**p value**
N patients	68 (58%)	50 (42%)	
Male gender (N,%)	52 (76%)	36 (72%)	0.6
Age (years, mean ± SD)	51.2 ± 5.6	50.6 ± 5.9	0.1
Foreign nationality (N,%)	1 (1.5%)	1 (2.0%)	0.8
HIV-1 non B subtype (N,%)	15 (22%)	7 (14%)	0.3
Risk factor (N,%)			
- parenteral	44 (65%)	27 (54%)	
- sexual	23 (34%)	21 (42%)	0.4
ART duration (years; median, IQR)	13 (9–16)	14 (10–16)	0.5
- NRTIs	13 (9–16)	14 (10–16)	0.6
- NNRTIs	4 (1–7)	4 (2–6)	1.0
- PIs	7 (5–9)	6 (5–12)	0.5
Past use of mono- or bi-therapy (N,%)	43 (63%)	35 (70%)	0.8
Previous AIDS events (N,%)	7 (10%)	10 (20%)	0.2
Serious non-AIDS events (N,%)	19 (39%)	7 (14%)	0.07
HBsAg pos (N,%)	9 (13%)	4 (8%)	0.1
Anti-HCV pos (N,%)	51 (75%)	30 (60%)	0.1
pVL (log_10_ cp/ml;median, IQR)	3.2 (3.2-4.8)	4.1 (3.2-8.9)	0.1
Aviremic pts (N,%)	53 (78%)	36 (72%)	
CD4+ cell count (median, IQR)			
- absolute (n/mmc)	658 (219–185)	558 (109–1219)	0.1
- percentage (%)	31 (24–53)	28 (11–52)	0.1
Nadir CD4+ cell count			
- absolute (median n/mmc;, IQR)	137 (90–616)	87 (27–382)	***0.005***
- percentage (%)	11 (7–27)	8 (2–32)	***0.01***

Legend: N = number, SD = standard deviation; IQR = InterQuartile Range; pVL: HIV-1 plasma viral load.

### Association with viro-immunological parameters

**Figure 1:**
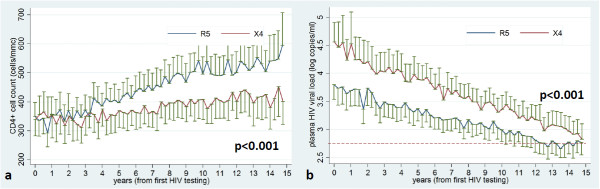
**Association of CRT with CD4 absolute count over time (a) and HIV cumulative viremia (b).** Legend: x axis (time) = years; y axis: **a**): CD4 = n/mmc, **b**): cumulative viremia = HIV-RNA log10 copy/years. The graph represents mean CD4 and HIV viral load (and standard errors) over time for R5 and X4 groups, predicted from *multivariate mixed models* including *patient* as “clustering” variable and *time* from first HIV testing (with quarters as unit of analysis), adjusting for baseline values (see Statistical methods). The red dashed line (Figure 1b) indicates the level of HIV RNA <500 copies/ml (2,69 log).

No differences were observed between R5 and X4 patients either in pVL value at time of analysis, or in the proportion of patients presenting an undetectable viral load. Similarly, both the absolute and percentage CD4+ cell count in the two groups were not statistically different, even if X4 patients presented a median number of CD4 about 100 cells lower when compared to R5. On the contrary, the presence of a X4 strain was significantly associated with the nadir CD4 cell count (both absolute, p = 0.005 and percentage, p = 0.01); moreover, it was strictly linked to the CD4 absolute count over time (p < 0.001) (Figure 1a). In addition, patients carrying an archived X4 also showed a greater cumulative incidence of positive viremia (copy/years) (p < 0.001) during the entire >15-year follow-up (Figure 1b).

### Association with antiretroviral therapy

Only five patients were naive to antiretroviral therapy (3 bearing an R5 virus and 2 a X4 virus); from information stored in our database, these five patients did not require therapy as they could be considered long-term non-progressors. An additional two patients (both R5) were not on treatment at time of analysis because of a voluntary therapy interruption. In the remaining patients, no association was noted between CRT and overall duration (years) of antiretroviral therapy, past exposure to mono/dual therapies, or duration of NNRTI or PI-based therapy.

### Markers of inflammation and aging

No differences were found between R5 and X4 patients regarding Il-6 levels (p = 0.7), levels of hsPCR (p = 0.5) and D-dimers (p = 0.6), nor was an association observed with IL-7 levels (p = 0.5) (Table 2).

**Table 2:** Lack of association of CRT with inflammatory markers in 118 patients

	**n < limit**	**β (X4 vs R5)**	**Lower limit**	**Upper limit**	**p value**
			**CI 95%**	**CI 95%**	
hsPCR (log)	62	0.501	−1.219	2.22	0.57
D-dimers (log)	0	0.280	−1.336	0.777	0.60
IL-6 levels (log)	23	0.022	−0.175	0.131	0.78
IL-7 levels (log)	89	0.259	−1.184	0.666	0.58

N: number of patients; limit: detection limit of the assay; β: coefficient of interval regression; CI: confidence interval.

Since inflammation marker measurements are subject to lower detection limits, the association of IL-6, IL-7, HsPCR, D-dimers and CRT was analyzed with interval regression by means of the intreg command Stata after log transformation (natural logarithm). None of the markers resulted significantly associated with CRT.

**Figure 2:**
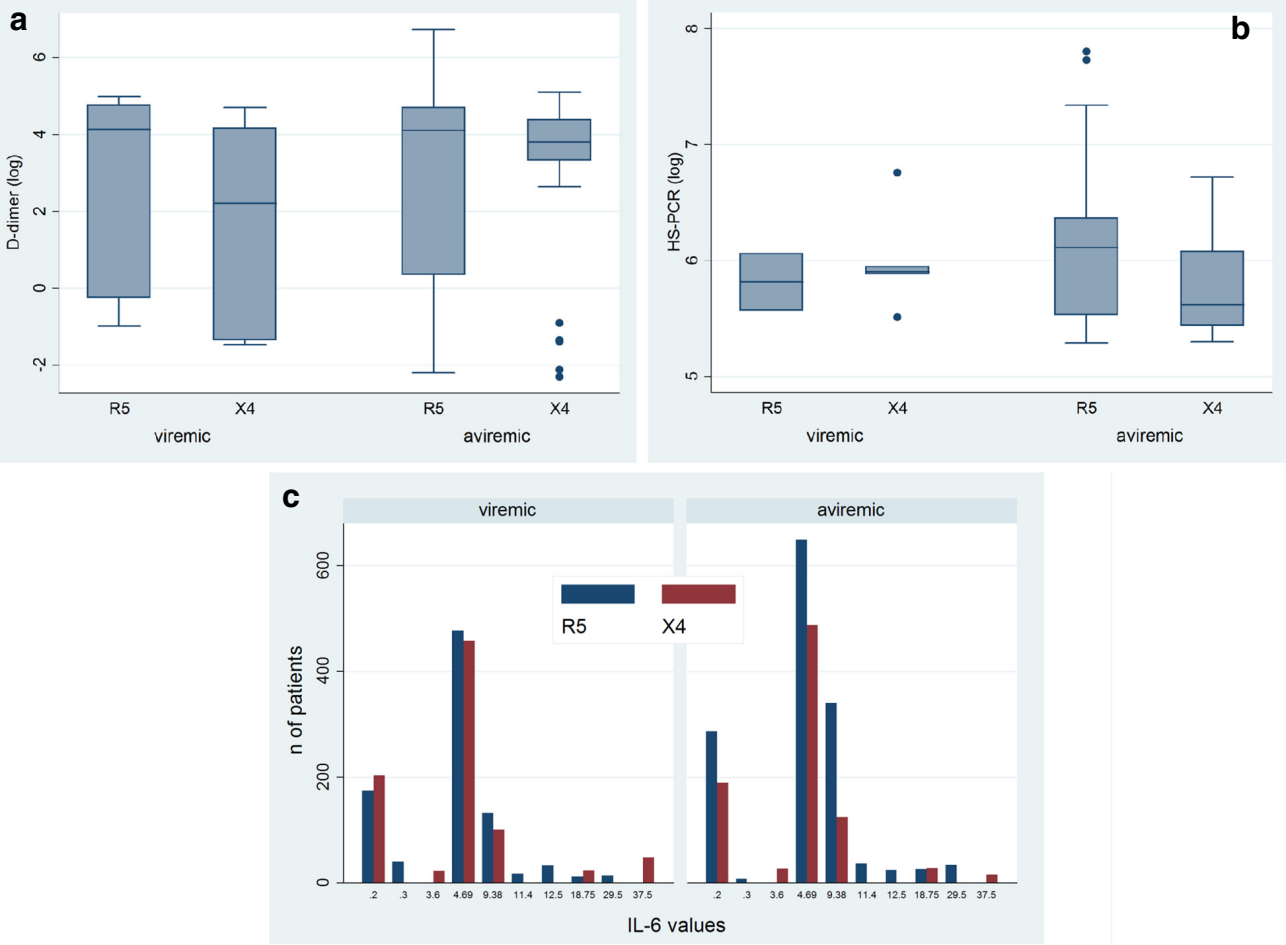
**Association of inflammation markers with CRT in viremic and aviremic patients.** Secondary adjusted analyses were performed to test whether markers were different according to CRT (R5 vs X4) and viremia (viremic vs aviremic), and both together (test for interaction). Results are showed as: **a**) and **b**): box and whiskers plot for log D-dimer (ng/ml) and log hs-PCR (ng/ml) (continuous variables), respectively, with breakdown for CRT, separately for viremic and aviremic patients; **c**) bar graph for IL-6 (pg/ml)(ordinal variable), with breakdown for CRT, separately for viremic and aviremic patients. No significant results were found.

Il-6, D-dimers and hsPCR levels were not correlated with the presence of detectable HIV-RNA at time of testing, nor with the cumulative viremia over time. Secondary adjusted analyses were performed to test whether markers were different according to CRT (R5 vs X4) and viremia (viremic vs aviremic) when tested for interaction, but no significant results were found (Figure 2) Moreover, none of the markers resulted associated with age. Only for IL-6 was a certain association found with the CD4 count at time of testing (β[per 100 CD4] = 0.023, 95% CI −0.05-0.0006, p = 0.056) and the cumulative CD4 count over time (β[per 100 CD4] = 0.002, 95% CI −0.004- -0.00012; p = 0.03). No biomarker demonstrated a correlation with the frequency of previous or current AIDS and non-AIDS related events.

### Analysis with different FPR cut-offs

In order to evaluate the potential impact of the chosen FPR cut-off on the final outcome, data were also analyzed setting the FPR at 10% and 5%; also in this case, no association was observed between X4 and R5 patients and levels of inflammation markers; once more, the only variable which resulted associated with CRT was the CD4 nadir, either when using a 10% FPR (both absolute, p = 0.002 and percentage, p = 0.004) or a 5% FPR cut-off (both absolute, p = 0.004 and percentage, p = 0.001).

## Discussion

In the pre-HAART era, many studies demonstrated that SI and/or X4 viruses emerged in approximately 40%–50% of patients progressing to AIDS, suggesting that these variants were associated with a worse clinical and viro-immunological outcome [8]. More recently, this percentage was found to range from 10% to 20% in naïve patient cohorts [31-33] and from 40% to 50% in multi-failed heavily-treated patients from maraviroc registrative trials [17-19,34], including a small proportion (1%-4%) of pure X4 and a large majority of R5/X4 (dual mixed, DM) strains.

Herein, we focused on patients surviving chronic HIV infection for at least 15 years with a long history of suppressed viremia under antiretroviral therapy in the majority, high CD4 median level, and no opportunistic infection at time of analysis. The results indicate that, also in this population, >40% of patients presented an archived X4 virus in proviral DNA, a percentage which is similar, or even higher, when compared to other recent reports [35,36] in suppressed patients with a shorter history of viremia suppression. Therefore, examining DNA for CRT determination should be recommended for all patients before prescription of chemokine inhibitors. Although the proportion of X4-carrying patients might be overestimated in DNA with respect to plasma [37], recent studies confirm that tropism switches under ART suppression are rare. Therefore, the current proviral DNA can be considered a good mirror of the patient viral quasispecies, including both recent or former replication variants [28,36].

Not only was a correlation with CD4 nadir evidenced, in agreement with previous studies [35], but X4 variants were significantly associated with exposure to higher cumulative viral loads and poorer immunological control over time, rather than with the viro-immunological state at time of testing. From the design of the study, it is not possible to resolve the pending question of whether X4 viruses emerged (and were archived in proviral DNA) as a consequence of the continuous replication and immunological impairment or, on the contrary, were responsible for disease progression in these patients. Even if several successive regimens might have masked the actual impact of therapy on CRT, according to other publications [35], the duration and type of antiretroviral therapy did not seem to influence CRT in our series. This finding also confirms our previous observation that, in patients treated by HAART and sequentially tested for CRT, the co-receptor shift is independent of virological success [15].

None of the baseline demographic and clinical characteristics, apart from CD4 and viral load trends over time, seem to influence the predominant strain archived in proviral DNA. In particular, even if the risk factor for HIV acquisition might be a determinant for CRT selection at time of transmission [38], it seems to lose its importance over time. A lower proportion of X4 strains has been observed among some non-B-subtypes, in particular subtype C [39], suggesting a possible association with an improved virological outcome for non-B patients when excluding racial and social variables, as recently described by some authors [40,41]; in the present study, however, non B-subtypes were rare and no definite conclusion can be drawn concerning the relationship between CRT and viral subtype. Moreover, the co-infection with other viruses, in particular with HBV and HCV, failed to demonstrate any association with CRT. In HIV-HCV co-infection, the env gp120 of HIV was supposed to interact with HCV E2 in triggering the apoptosis mechanisms [42] to modulate the biology of human hepatic stellate cells which play a key role in the fibrosis pathogenesis [43], and to enhance the replication of HCV through the engagement of extracellular coreceptors on hepatocytes [44]. In this study, a slightly higher proportion of R5 patients was observed among the HCV-positive patients when compared to negatives, but without statistical significance. If R5 and X4 strains can be differentially involved in determining viral persistence and fibrosis progression in HCV positive patients should be object of further research. No differences in AIDS-related and serious non-AIDS events were observed in the two groups, but, as only long surviving patients were included in the study, pathological events leading to death were not considered and therefore we cannot exclude that severe AIDS occurrences might be variously distributed in X4 and R5 patients.

Many reports indicate that HIV-infected patients develop an inflammation and hypercoagulation state which is involved in the aging mechanisms. Surrogate serological markers such as IL-6, D-dimers and hsPCR have been demonstrated to be good prognostic markers in HIV-infected patients and are generally associated with elevated HIV-1 viral loads [23-26]. It has been hypothesized that X4-patients present an accelerated rate of disease progression compared to R5. In fact, also in our patient cohort aging with HIV, the presence of X4 strains was associated with a poorer immunological and virological control over time; therefore, we evaluated the hypothesis that CRT might be correlated with a worsened inflammation state as measured by the above mentioned surrogate markers. However, HsPCR, D-dimer and IL-6 s levels did not differ between the two groups of R5 and X4 patients. Therefore, in our experience, CRT did not seem to affect the inflammation state in patients aging with HIV, at least when measured by means of surrogate markers. None of the other patient characteristics was predictive of higher levels of IL-6, HsPCR and D-dimers in our cohort, except for a lower current and cumulative CD4 cell count for IL-6, which would seem to suggest, according to other studies [45], that immune-depression is an essential driving force for inflamm-aging in HIV positive patients. A limitation of our findings is represented by the fact that surrogate markers do not show any difference according to age or, more importantly, between patients with active HIV replication compared to those with undetectable viraemia. This is most likely due to the particular characteristics of our population which included patients with a narrow age range (median 50, IQR 47–53 years) and only a limited number of subjects with detectable HIV-RNA (25%). Moreover, although IL-7 was demonstrated to induce viral evolution of X4 viruses *in vitro*[30], no association between current IL-7 levels and the presence of archived X4 variants *in vivo* was observed in the present study; however, it cannot be excluded that lymphopenia-induced IL-7 production might have previously favored the R5 to X4 switch at previous timepoints during the patient’s history, but this association was not apparent at time of analysis.

Lastly, the issue concerning the clinically relevant FPR cut-off for CRT attribution based on geno2pheno is still controversial; herein, according to the 2011 European Guidelines [46], the 20% FPR cut-off was chosen since interpretation was based on a single DNA amplification and sequencing; however, similar results were also obtained when data were analyzed using a 10% and a 5% cut-off, excluding the potential impact of this choice on the final outcome of the analysis.

This is one of the few studies regarding CRT in patients aging with HIV (all patients living at the time of study design have been included) and the first, to our knowledge, which investigates the relationship between the CRT and state of inflammation. However, the study presents several limitations. All the above reported lack of associations might be due to a sample of insufficient size to detect small effects, particularly in subgroup analyses, interactions and multivariable adjustments. Furthermore, due to its retrospective design, some relevant baseline characteristics might not have been measured: for instance, for some of the older patients, baseline viral load was not available (before 1995); in fact, cumulative viral load is probably underestimated for all patients. Also, repeated and prospectively collected measurements of inflammation markers were not performed, and we can only estimate the influence of point determination of these parameters. Finally, as already stated, patients have been “naturally” selected on the basis of their vital status at time of CRT testing, and it must be kept in mind that patients with a shorter HIV history might differ from this highly selected population.

## Conclusions

A significant proportion (42%) of patients with a >15 year history of HIV infection had an archived X4 virus. This presence was clearly associated with a longer duration of exposure to positive viremia and with a poorer CD4 trend over time when compared to R5, independent of the duration of antiretroviral treatment, even if a causal relationship could not be established. The CRT does not seem to influence the inflammation rate of patients aging with HIV.

## Abbreviations

HIV-1: Human immunodeficiency virus type 1; CRT: Co-receptor tropism; CXCR4: C-X-C chemokine receptor type 4; CCR5: C-C chemokine receptor type 5; X4: CXCR4-using virus (non-R5); R5: CCR5-using virus; DM: Dual-mixed virus; FPR: False positive rate; pVL: Plasma viral load; HAART: Highly active antiretroviral therapy; ART: Antiretroviral therapy; CDC: Center for Disease Control.

## Competing interests

The authors declare that they have no competing interests.

## Authors’ contributions

AS, LM and GA conceived the study and participated in its design and coordination. AS drafted the manuscript. GB and NL were responsible for data collection and participated to the study design. GP and AL carried out the viral genotyping and sequencing. AV carried out the immunoassays. LS performed the statistical analysis; LS, LM and GB participated in the interpretation of data and revised the paper critically. All authors read and approved the final manuscript.

## Pre-publication history

The pre-publication history for this paper can be accessed here:

http://www.biomedcentral.com/1471-2334/13/220/prepub
